# Exploratory Study of Fecal Cortisol, Weight, and Behavior as Measures of Stress and Welfare in Shelter Cats During Assimilation Into Families of Children With Autism Spectrum Disorder

**DOI:** 10.3389/fvets.2021.643803

**Published:** 2021-09-06

**Authors:** Gretchen K. Carlisle, Rebecca A. Johnson, Colleen S. Koch, Leslie A. Lyons, Ze Wang, Jessica Bibbo, Nancy Cheak-Zamora

**Affiliations:** ^1^University of Missouri College of Veterinary Medicine Research Center for Human-Animal Interaction, Columbia, MO, United States; ^2^Animal Behavior Services, Lincoln Land Animal Clinic, Jacksonville, IL, United States; ^3^Department of Veterinary Medicine and Surgery, College of Veterinary Medicine, University of Missouri, Columbia, MO, United States; ^4^University of Missouri Department of Educational, School and Counseling Psychology, Columbia, MO, United States; ^5^Benjamin Rose Institute on Aging, Center for Research and Education, Cleveland, OH, United States; ^6^University of Missouri School of Health Professions, Columbia, MO, United States

**Keywords:** cat stress, autism spectrum disorder, children, shelter cats, cat adoption

## Abstract

**Background:** Cats are a common companion animal (CA) in US households, and many live in families of children with autism spectrum disorder (ASD). The prevalence of ASD is one in 54, and many children have behavior challenges as well as their diagnostic communication disorders.

**Objective:** Benefits of CAs for children with ASD have been identified, but little is known about the welfare of CAs in these homes. This study explored the welfare of cats (*N* = 10) screened for ideal social and calm temperament using the Feline Temperament Profile (FTP) and adopted by families of children with ASD.

**Methods:** Cat stress was measured using fecal cortisol, weight, and a behavior stress measure (cat stress score). Measures were taken at baseline in the shelter, 2–3 days after adoption, and at weeks 6, 12, and 18.

**Result:** Outcome measures suggested the adopted cats' stress levels did not increase postadoption; however, the small sample size limited analytical power and generalizability.

**Conclusion:** This study provides preliminary evidence for the success of cat adoption by families of children with ASD, when cats have been temperament screened and cat behavior educational information is provided. Further research is warranted to confirm these findings.

## Introduction

The most common companion animal in US homes is the housecat with over 86 million living in American homes (1). Among these homes are many families of children with autism spectrum disorder (ASD). One in 54 children are identified as having ASD, and symptoms for these children include social, communication, and other behavior challenges (2). These behavior problems could pose potential challenges for the well-being of cats. Children with ASD are shown to benefit from the presence of companion animals (CAs) (3–5). However, to our knowledge, no specific studies have been conducted on the welfare of cats residing in homes of children with ASD.

Cats adapt to and respond positively to consistent, safe predictable environments. Cats may exhibit problem behaviors when placed in stressful situations, such as, entering a new family (6). Households with children can be stressful; families of children with ASD may represent an especially stressful home environment. Approximately 70% of children with ASD are reported by their parents to have had severe temper tantrums at some point and 60% of those within the severe tantrum group were reported to continue to have tantrums on a daily basis (7). Loud sounds accompanying tantrums could present a challenge for cats exposed to this noise. Amat et al. (8) reported cats with noise-related phobias are more likely to respond with redirected aggression toward humans. Cats routinely subjected to unpredictable child behavior and noise may have increased stress. The behavior changes observed, secondary to stress, may mimic medical conditions such as anorexia, vomiting, and diarrhea (9) with resultant weight loss indicating a decline in health (10).

Screening cats for calm and social temperament for adoption by families of children with ASD and identifying cat stressors may be key to promoting cat welfare in home introductions. The Feline Temperament Profile (FTP) has been validated as an objective measure of cat temperament. Increased stress, which may result in undesirable behavior, leads to a physiological response of increased cortisol in cats (11, 12). Cortisol can be measured in cat feces, providing a non-stressful and reliable method of sample collection (13). Cats often need a minimum of a 12-week time period to adjust to a new environment (6). Therefore, assessing both behavioral and physiological measures (13) for at least 12 weeks should be adequate to identify welfare issues within new home environments.

The aim of this study was to explore the stress and welfare of cats in families of children with ASD. The following hypothesis was posited: After a transition period, shelter cats screened for social and calm temperaments introduced into the families of children with ASD will not demonstrate significant signs of stress (i.e., increased fecal cortisol levels, weight loss, or stress behaviors).

## Materials and Methods

### The Experiment

This study was approved by the Animal Care and Use Committee of the University (protocol #9583). This study was nested into a randomized controlled trial with families of children with ASD adopting shelter cats (14). A repeated measures design with data collection at baseline, 2–3 days after cat adoption, and weeks 6, 12, and 18 was conducted. Parents in the adopting families were also contacted by study staff 3 weeks after adoption to inquire about cat acclimation and to offer educational support, if needed. Results on the child arm of the study, including child-cat interaction and bonding go beyond the aim of this study and were published separately (14). Findings in the child arm indicated that cat adoption might be beneficial for some children with ASD.

### Animals

Cats were screened from two participating animal shelters and required to reach a score of 20 or greater on the FTP to be eligible for adoption. Any cat attempting to bite during the FTP assessment was excluded, regardless of the FTP score. Cats were required to be between the ages of 10 months to 4 years old (based on estimated ages by shelter staff). All cats were spayed or neutered, current on vaccinations, and without health-related issues at the time of adoption.

Participants (hereafter referred to as families) included parents and their child with ASD aged 6–14 years old and not currently living with a cat in their home. Families were excluded if anyone living in their home had cat allergies or acknowledged a history of aggression toward an animal. Families were required to obtain approval to adopt a cat through the standard adoption protocol of the animal shelter where they chose their cat. The study staff provided written and verbal cat care information to the families, including, specific techniques regarding the introduction of a cat into a new environment and how to help cats with life stressors, litter box training, and engaging cats in play, based on recommendations and guidelines developed by The Ohio State Indoor Pet Initiative (15). Each animal shelter also provided verbal instructions on cat adoption.

Once adopted, families were required to maintain their cat indoors only throughout the study and were responsible for all the healthcare of their cat following adoption from the animal shelter. Adopting shelters fed a variety of cat foods as free feeding without restrictions. The study families were supplied with cat food, and cats were allowed free feeding in their adoptive homes. Families were also supplied with cat litter for the duration of the study. Additional supplies families permanently kept included a litter box, cat carrier, toys, and a scratching post with a two-tier level climbing tree, which included a small covered “apartment” to serve as a safe space for their cat. Parents were required to agree to contact the study staff if their child showed any evidence of aggression toward the cat or purposefully injured the cat. If any cat scored a 7 on the Cat Stress Score (CSS), indicating high stress, the study staff would review the educational information with the parents, and the veterinary behaviorist on the study team was available to consult with the family. Study staff were available through telephone contact throughout the study if families had any concerns or questions.

### Instruments and Data Collection

The FTP measures cat temperament, sociability, flexibility, aggressiveness, and cat-human match utilizing scores compiled from 10 items which each include “acceptable” and “questionable” behaviors (16). Each item includes a tester directive such as “while talking to the cat, begin to stroke the cat along the head, back and sides.” The tester then observes the cat for behaviors such as “rubs against legs or hand” as an example of an acceptable behavior or “bites/attempts to bite hand” as an example of a questionable behavior. One point is allowed for each behavior observed. The FTP is a reliable instrument with Siegford et al. (16) finding no statistically significant difference in cats scored as acceptable before adoption, and at follow-up evaluation, 3 and 6 months postadoption [*F*_(3, 76)_ = 1.29; *p* = 0.28]. The FTP provided the measure of inclusion criterion for the cats in this study. Shelter staff were requested to identify calm cats they would typically consider suitable for a potentially noisy household with children; neither shelter utilized an objective behavior scoring protocol. Cats referred by the shelter staff were then screened by study staff using the FTP to further assess for adoption suitability by study families.

After the families selected their cats for adoption, the cats were held at the shelter until shelter staff were able to collect a fecal sample. Shelter staff stored the fecal samples in freezers measuring at least −8°C and contacted study staff for pick up. Baseline cat weight and CSS was then obtained by study staff and fecal sample transported in a thermal cooler with ice packs to a laboratory freezer measuring −80°C. After cat baseline data were collected, families were notified their cat was ready to be picked up for adoption. All remaining data collection occurred in the family homes.

The CSS served as the behavior outcome measure. The CSS is a measure of cats' stress in novel environments (17). The cat is rated on a 7-point scale (1 = “fully relaxed” to 7 = “terrorized”) for different body parts (e.g., body, belly, legs, etc.) and behavior, including vocalization and activity. For example, a body observation score for “fully relaxed” includes the descriptive of “laid out on back or side” and a score of “terrified” includes the description of “crouched directly on top of all fours, shaking.” The reliability coefficient of the CSS was 0.9 in cats housed in boarding catteries (17).

Study staff were regularly trained on administration of the FTP and CSS instruments and reassessed throughout the study. Study staff were trained in behavior rating and data collection by a veterinarian practicing in small animal medicine. This training included videotapes of cat behaviors rated by a board-certified veterinary behaviorist. Videotapes were viewed by team members who rated cat behaviors. If ratings did not reach 90% or greater agreement with the behaviorist's ratings, study staff were required to retrain with the veterinarian until their rating reached 90%. In addition to this, all seven staff raters were required to retrain and their ratings to reach 90% agreement every 6 months during the study.

Cat body weight was measured as a stress indicator in cats, since eating less has been observed in cats under stress resulting in weight loss (10). Cat weights were measured using the Redmon Precision digital pet scale Model 7475 in the shelters and families' homes. Cats were weighed by study staff in homes after CSS score was obtained by placing it directly on the scale by gently picking the cat up or luring the cat with a treat onto the scale. If the cat remained hidden after 5 min and was not visible enough to obtain a CSS assessment, no score was given. At this time, the owner placed the cat in the carrier so a weight could be obtained. If the parent requested assistance to place the cat in the carrier, the study staff assisted by slowly approaching and gently placing the cat into the carrier. After weighing the cat the carrier door was opened to allow the cat to leave the carrier.

Cat fecal cortisol samples served as a physiological stress measure. Adrenocortical activity can be reliably measured through fecal cortisol in cats (18). Cortisol metabolites are stable in fecal samples at room temperature for up to 12 h before freezing (19). The fecal samples can be stored in a household freezer after defecation and transported in a thermal cooler to the laboratory for storage at −20°C until prepared for analysis (20). Fecal glucocorticoid levels were determined using a commercially available corticosterone radioimmunoassay (DA Corticosterone kit, ICN MP Biomedicals), which has been previously validated for domestic cats (18). The lower detection limit of the assay was 0.26 ng/ml and upper detection limit was 20 ng/ml. The assay was performed according to the manufacturer's protocols with the exception that standard diluent was added to the fecal extracts and fecal extraction buffer (containing 50% methanol) to the standards. Concentrations were determined as nanograms per milliliter and then divided by the dry weight of extracted feces to give the results as nanograms per gram feces. All samples were assayed in duplicate. Assay accuracy was assessed by adding a known amount of corticosterone to four fecal extracts that contained low values of glucocorticoids.

Study staff were trained in the non-invasive technique of obtaining, transporting and storing of the fecal samples and weighing the cats. Cat fecal samples were collected by parents who were taught the proper procedure by study staff and instructed to collect a sample within 24 h before the study site visit. Home freezers all functioned at a minimum of −8°C. If no fecal sample was collected by parents, study staff instructed the parents to call the study office when a sample was collected (within three days after visit) and study staff returned and picked up the sample. Samples were transported by study staff in a thermal cooler with ice packs to the laboratory freezer. Samples were shipped using dry ice in batches to the analyzing laboratory.

For fecal hormone extraction, approximately 0.5 g of wet fecal material was weighed then shaken overnight in 5 ml of a modified phosphate-saline buffer containing 50% methanol (21). Liquid extracts were decanted, and solids were removed through centrifugation at 4,000 × *g*. Supernatants were then frozen at −80°C until assay. Fecal solids were placed in a drying oven overnight at 80°C before the assays were conducted. The mean intra assay variation of duplicate samples was 9.7%; the mean inter assay variation of two quality control pools was 7.6%. For assay validation, fecal extracts were tested for linearity by diluting four samples that contained high levels of glucocorticoids by 1/2, 1/4, 1/8, and 1/16 with extraction buffer. Serial dilutions of fecal extracts averaged 90.9 ± 2.6% of expected values. Addition of known amounts of hormone at three dosage levels resulted in the recovery of 101.3 ± 1.7% of added corticosterone.

### Statistical Analysis

Descriptive statistics and repeated measures analysis of variance (ANOVA) were used for each of the variables: cat fecal cortisol, cat weight, and CSS. The variables seemed to be normally distributed based on the skewness and kurtosis statistics. A significance level of 0.05 was considered statistically significant for all analyses.

## Results

### Cat Descriptives and Measures

The shelter staff identified 235 cats as calm and acceptable for adoption by a noisy family. Only 80 of the shelter staff assessed cats met the study criteria of an FTP of 20 or greater for a 34% pass rate. Participating families were allowed to choose a cat from among those passing the FTP criteria. Cats passing the FTP included 46 female and 34 males with a mean age of 21.8 months, and 62 were spayed/neutered. Passing cats had a mean FTP score of 22.4. Adopted cats are described in Table 1. Ten cats, seven females and three males were adopted by families. The shelter staff described nine of the cats as domestic shorthair and one as a Russian Blue (cat 1). Cat ages were estimated from 10 months to 4 years with a mean of 18.4 months, and all cats were spayed or neutered before adoption. The FTP acceptable scores ranged from 20 to 29 with a mean of 24.5 (Tables 1, 2). The raw scores for cat fecal cortisol, weight, and CSS at each time point are in Table 1. Table 2 provides the sample means for the demographics as well as all outcomes. Two cats were relinquished to the adopting shelters; one was reportedly due to failure to bond with the child with ASD and house soiling at week 6, and the second reportedly due to undesirable vocal behavior of the cat at week 12.

**Table 1:** Individual cat characteristics, signalment, temperament, and stress evaluations.

**Cat characteristics**					**Fecal cortisol (ng/g)**					**Weight (kg)**					**Stress score**				
**ID**	**FTP A**	**FTP Q**	**Sex**	**Age**	**BL**	**T1**	**T2**	**T3**	**T4**	**BL**	**T1**	**T2**	**T3**	**T4**	**BL**	**T1**	**T2**	**T3**	**T4**
1	26	0	F	0.83	759.4	479.6	26.3	388.6	60.3	2.82	2.89	2.89	3.63	3.69	4.0	5.0	2.0	4.0	2.0
2	26	0	F	1	39	54.8	56	107.1	–^d^	3.63	3.40	3.97	3.97	4.20	2.0	2.0	2.0	1.5	1.0
3	22	2	F	2	52.8	166.2	108.5	213.5	–^b^	4.37	4.20	4.37	4.42	–^b^	2.0	3.0	2.0	1.5	–^b^
4	25	2	M	1	100.1	94.7	129.8	56.3	85.3	4.37	4.48	5.28	6.35	6.30	3.0	2.5	3.0	3.0	–^e^
5	26	1	F	1	641.5	118.1	127.5	146.7	116.3	3.80	3.52	3.97	4.82	4.79	3.0	1.0	2.0	–^e^	1.0
6	24	0	M	2	305.1	219.5	322.4	116.4	512.7	4.42	4.31	4.71	5.28	4.99	2.0	2.5	3.0	2.0	1.5
7	23	3	F	1	421.7	73.2	–^a^	–^a^	–^a^	2.89	3.18	–^a^	–^a^	–^a^	2.0	1.5	–^a^	–^a^	–^a^
8	20	0	F	1	826.3	707.2	282.4	205.4	–^a^	2.45	3.01	4.08	–^a^	–^a^	4.0	2.5	2.0	–^a^	–^a^
9	29	1	F	1.5	–^c^	210.3	89.1	282.7	139.5	–^c^	2.61	2.89	2.95	3.12	–^c^	2.0	1.5	2.0	2.0
10	24	0	M	4	756.5	124.2	183.9	90.1	173.1	10.63	4.94	5.67	5.73	5.84	1.0	–^e^	2.0	3.0	1.5

*FTP A, number of acceptable feline temperament profile behaviors; FTP Q, number of questionable feline temperament profile behaviors; BL, baseline; T1, 2 to 3 days following adoption; T2, 6 weeks following adoption; T3, 12 weeks following adoption; T4, 18 weeks following adoption*.

a *Missing data due to relinquished cat*.

b *Missing data due to participant move out of area*.

c *Missing data due to shelter failure to collect before adoption*.

d *Missing data due to participant inability to collect*.

e *Missing data due to cat in hiding*.

**Table 2:** Sample characteristics, signalment, temperament, and stress evaluations.

**Characteristics**				**Fecal cortisol (ng/g)**					**Weight (kg)**					**Stress score**				
***M*** **(SD) or %**				***M*** **(SD)**					***M*** **(SD)**					***M*** **(SD** ***)***				
**FTP A**	**FTP Q**	**Sex (F)**	**Age**	**BL**	**T1**	**T2**	**T3**	**T4**	**BL**	**T1**	**T2**	**T3**	**T4**	**BL**	**T1**	**T2**	**T3**	**T4**
24.5	0.9,	70%	1.5	433.6	224.8	147.3	178.5	181.2	3.72	3.67	4.2	4.63	4.72	2.6	2.4	2.2	2.4	1.5
(2.5)	(1.1)		(1.0)	(323.3)	(208.6)	(99.3)	(105.9)	(167.2)	(0.86)	(0.77)	(0.95)	(1.13)	(1.13)	(1.0)	(1.1)	(0.5)	(0.9)	(0.4)

*FTP A, number of acceptable feline temperament profile behaviors; FTP Q, number of questionable feline temperament profile behaviors; BL, baseline; T1, 2 to 3 days following adoption; T2, 6 weeks following adoption; T3, 12 weeks following adoption; T4, 18 weeks following adoption*.

### Fecal Cortisol

Raw scores for all cat fecal cortisol measures are plotted in Tables 1, 2 provides sample means for this outcome. Only five cats had complete fecal cortisol data collected at all five time points, thus were used in analysis. Mauchly's test of sphericity indicated that the assumption of sphericity had not been violated, χ^2^(9) = 13.68, *p* = 0.218. A statistically significant time effect on fecal cortisol [*F*_(4, 16)_ = 3.45, *p* = 0.032] was present. More specifically, fecal cortisol at week 12 was statistically significantly lower than at baseline (*p* = 0.032), although the values at days 2–3 and week 6 were nearly statistically significantly lower than at baseline (*p* = 0.065 for comparison between days 2–3 and baseline, and *p* = 0.089 for comparison between week 6 and baseline).

### Cat Weight

Raw scores for all cat weights are plotted in Tables 1, 2 provides sample means for this outcome. Three cats (cat 3, cat 7, and cat 8) had incomplete data. Mauchly's test of sphericity indicated that the assumption of sphericity had not been violated, χ^2^(9) = 10.12, *p* = 0.396. A statistically significant time effect was observed for cat weight [*F*_(4, 20_) = 19.96, *p* < 0.001]. After an initial nonsignificant drop in weight from baseline (at shelter) to weight at 2 to 3 days after adoption, the average cat weight increased at weeks 6 (*p* = 0.008) and 12 (*p* = 0.027), and cats maintained this weight at week 18.

### Cat Stress Score

Raw scores for CSS are plotted in Tables 1, 2 provides sample means for demographics and outcomes. Only three cats had complete CSS data collected at all five time points. Missing data included four instances with cats that were in hiding and observation without intervention was unavailable. In one case, a hiding cat occurred on the first visit, 2–3 days after adoption; in another case, it occurred on a visit following the family's return from a 2-week vacation. Lack of CSS data also occurred from two cats that were relinquished before the end of the study and one cat that moved out of state before the final data collection visit. The sphericity assumption was not applicable with the small sample size. There was no statistically significant time effect on CSS [*F*_(4, 8)_ = 1.82. *p* = 0.218].

## Discussion

Our exploratory findings suggest the cats in the study, screened for calm temperament using the FTP, acclimated to their adopted homes of families of children with ASD. No empirical literature measuring the acclimation of cats into families with typically developing children could be identified in order to compare results. The FTP, found to be reliable in screening shelter cats for adoption (22), provided an objective screening measure for temperament of cats by identifying cats that were calm, sociable, and without aggressive behaviors during the multiple handling procedures of the assessment. The American Society for the Prevention of Cruelty to Animals (23) reported only 15% of 555 shelters responding to their survey utilized a written score to evaluate cat social skills. In this study, only 34% of cats selected by shelter staff passed the screening criteria to qualify for potential adoption. The utilization of a reliable and objective temperament screening tool by shelter staff may aid in decreasing the relinquishment/return of cats to shelters. Utilizing a temperament screening instrument may aid shelters in helping adoptees to identify a calm cat, which could be particularly important for families of children with ASD. While information on the benefits of dogs for children with ASD appears more often in the literature, cats may also provide benefits (24) when the cat is a good temperament match with the family. Further research could shed light on the value of this assessment procedure in reducing cat relinquishment vs. the time it would take to train and re-assess shelter staff and conduct the FTP screenings.

In addition to screening for temperament, written and verbal educational support was provided to aid families in the adoption process. The education of adult owners and understanding of cat behavior has been found to improve the welfare of cats in homes (25). Of the two cats who were relinquished, neither case was due to aggression; however, one was related to house soiling, which is a common behavioral reason for cat relinquishment (25–27). Therefore, the FTP was successful in identifying calm cats without aggression. Unfortunately, there is no way to predict if an animal will bond with a particular individual. Since the families did not want to pursue help through our veterinary behaviorist, insufficient information was available to determine if the lack of bonding, house soiling, and excessive vocalization in the relinquished cats were due to household stress or other unidentified medical conditions. The educational support, along with supplies including a climbing tree with a scratching post and “apartment” to provide the cat with a private space, which were all provided at the time of adoption, may have also aided the welfare of cats in our study, along with screening for calm temperament.

Elevations in stress may result in behavioral problems leading to relinquishment (28). In a previous study, no significant differences were identified in fecal cortisol levels of cats in shelters compared with their levels after adoption (29). In that study, adoptive owners collected an average of 10 fecal samples over a period of 3 months beginning an average of 3 months after the arrival of their cat (effect size = −0.37, *N* = 15, *p* = 0.068). In Fukimoto et al. (29), it is unclear whether the lack of difference in stress was due to similar stress in the adoptive homes compared with the shelter (i.e., environment) or the adaptability of the cats (i.e., temperament). Cat stress can be very difficult to measure in both the shelter and home environment. Fear and anxiety can result in aggression and the mislabeling of a gentle but fearful cat as an aggressive cat; likewise, a fearful but unfriendly cat may also be mislabeled as calm. Stress can also lead to medical problems as well as behavior problems (30). The potential for environmental stress is high when an animal is placed in a home with unpredictable human interactions. We expected cats to be stressed at the shelter and immediately after adoption; however, we hypothesized the stress would diminish over time. Stress was mitigated in our study through screening both the cat and the home prior to adoption, the education of the owner, as well as environmental enrichment including a safe hiding place for the cat. The reduction in fecal cortisol, as well as no reports of aggression or cystitis [a common sequalae in stressed cats (9, 31)], demonstrates the cat's welfare concerns were adequately addressed in potentially stressful environments.

Several families in this study had unique environmental changes that may have impacted cat stress. For example, in the case of cat 1, which had an elevation in FC on week 12, the parent reported the cat received a new microchip 2 days before the study visit. In addition to this, the parent of cat 1 reported a new significant other who began visiting the house with a dog that the cat reportedly “doesn't like.” In the case of cat 3, which also had an elevation in FC on week 12, the child was preparing to move out of state to live with the other parent in the next few days, and this may have created a more chaotic environment with packing and the potential stress of the people in the household. Cat 6 had fluctuations in FC throughout the study. This family included the child with ASD and three siblings under the age of 4 years. One younger sibling was observed to roughly pick up the cat during a visit. The cat wriggled free and jumped down, and the mother reminded the child to be gentle. The child with ASD reportedly had a strong bond with cat 6; however, the busyness of the environment may have been stressful for the cat. Cat 9 had an elevated FC on week 12, and the parent reported that the cat had escaped outside the home in the last few days before the visit but was now back inside.

Cats in our study gained weight after adoption and maintained their weight from 12 to 18 weeks. Buffington (32) reported an increase in stress-related behaviors such as decreased appetite in cats exposed to unusual environmental events. While we did not measure the frequency of behavioral outbursts of the children with ASD in our study, these typically are common occurrences and would have created a potentially stressful environment for the cats. Despite the environment, the initial increase in weight and maintenance of their new weight by cats in this study suggests fear and anxiety did not result in anorexia or decreased appetite, indicating the welfare of the cats in the study was adequate.

Because the sample size for these analyses is very small, while CSS scores overall decreased over time, that change is not statistically significant. Only three cats included a complete data collection set. Data collection for the CSS was particularly challenging with cats that hid on arrival of study staff, especially during the first data collection visit at 2–3 days postadoption. While a cat in this type of hiding would indicate possible fear and stress, accurate scoring was not possible without observation thus resulting in missing data points. Establishing a standard stress score for cats in hiding would have aided in minimizing missing data and would improve findings in future research. An additional measure of asking parents to video the cats between data collection visits might also have added to the understanding of cat welfare in the adoptive homes and would be a valuable addition to future research.

Of the two cats relinquished, the first was at week 6 and the FTP score of this cat was 23 acceptable and 3 questionable (when steady pressure pull of tail: struggles to escape and hisses; when placed on lap: jumps off lap). The parent reported the cat was not bonding well with the child and repeatedly eliminated outside the litter box. Coaching regarding cat behavior was offered by study staff at a 3-week follow-up call, along with an offer of a telephone consult with the veterinary behaviorist on the team, which the parent declined. Based on the FTP score of this cat, cats with any questionable scores might not be a good match for busy/noisy families, even if the cat's total FTP score is 20 or greater. The second cat was relinquished at week 12. The parent reported the child liked the cat, but the cat was vocal during the day while the child was at school and she did not like this behavior. Study staff offered educational behavior support, which was declined by the parent. A third cat was lost to follow-up when the child moved out of state to live with a different parent. Of note is the child took the cat along to the new home.

There are a number of limitations in this exploratory study which minimize the ability to generalize the findings. The original research protocol included a desired sample size of 64 cats. Despite rigorous recruitment techniques including extending the recruitment timeline to over two years, this did not aid in increased numbers of participants. The extended timeline resulted in a turnover of shelter volunteers/staff and one cat being adopted by the family before baseline data was collected. Regular ongoing volunteer/staff training at shelters in future studies would maximize communication with the study staff and minimize missing data. Recruitment efforts also included expanding the geographic area of recruitment up to 125 miles from the study site, sending recruitment flyers by mail, and telephone calling of 534 parents meeting the inclusion criteria from a mid-western autism center database of parents willing to be contacted for possible participation in research. Future studies might benefit from a recruitment site based in a larger metropolitan area. While we can infer from our findings the selection of cats with a FTP score of 20 or greater were likely to be associated with less stress in cats during adjustment, for ethical reasons, cats with lower scores were not selected for adoption by families in our study. Thus, there is no comparison of adopted cats not passing our FTP criteria. This point is moot if cat adoptions are to succeed, every advantage should be in place to provide potential adopters with cat temperament information to aid in a successful match for adoption.

Challenges for the families included an adoption process with two visits to the shelter and fecal sample collection. Despite reminder phone calls from study staff on the day before visits, study staff had to return on multiple occasions to collect samples the next day as parents reported not having a sample ready. Specific reminders during the phone call to collect a fecal sample, rather than just a reminder for the study visit may improve compliance in future studies. However, we acknowledge that even with the incentives provided such as waiver of shelter adoption fees, and cat supplies along with food and litter throughout the study, expecting busy families to collect specimens and have them available for pick up by study staff may be unrealistic.

The small sample in this study did not allow for comparisons of children with differing levels of severity of ASD. Thus, the highly variable symptoms and behavior of each child with ASD eliminated the ability to control for the environment; however, following the families for 18 weeks allowed time for the cats in each “case” to adapt to their environment. Another limitation was not including families with typically developing children for comparison. Future research would benefit from including typically developing children as a comparison group of cat adopting families.

## Conclusions

Screening of shelter cats using the FTP aided in identifying cats with a calm social temperament. Problem behaviors are common among children with ASD, and these may be potentially stressful for cats, especially those with a fearful or timid temperament. Cats adopted in this small exploratory study did not show signs of increased stress after assimilation into their new families. Matching calm cats and providing education to families with a highly variable environment may have aided the acclimation of cats in this study. Due to the small sample size it is not possible to make generalizations about cats adopted by families of children with ASD; however, the methodology provided by this study provides a starting point for future research to explore this important issue for cat welfare.

## Data Availability Statement

The datasets presented in this article are not readily available because Data is owned by the University of Missouri and not publicly available. Requests to access the datasets should be directed to carlislegk@missouri.edu.

## Ethics Statement

The studies involving human participants were reviewed and approved by University of Missouri Institutional Review Board. Written informed consent to participate in this study was provided by the participants' legal guardian/next of kin. The animal study was reviewed and approved by University of Missouri Animal Care and Use Committee. Written informed consent was obtained from the owners for the participation of their animals in this study.

## Author Contributions

GC, RJ, JB, and LL: Conceptualization. GC, RJ, and JB: Methodology. GC, RJ, LL, and ZW: Formal analysis. GC: Investigation, Writing—original draft preparation, Project administration. LL, CK, and NC-Z: Resources. GC and ZW: Data curation. GC, RJ, CK, LL, ZW, JB, and NC-Z: Writing—review and editing. GC and RJ: Funding acquisition. All authors have read and agreed to the published version of the article.

## Conflict of Interest

The authors declare that the research was conducted in the absence of any commercial or financial relationships that could be construed as a potential conflict of interest.

## Publisher's Note

All claims expressed in this article are solely those of the authors and do not necessarily represent those of their affiliated organizations, or those of the publisher, the editors and the reviewers. Any product that may be evaluated in this article, or claim that may be made by its manufacturer, is not guaranteed or endorsed by the publisher.
